# The Relationship between Affect Integration and Psychopathology in Patients with Personality Disorder: A Cross-Sectional Study

**DOI:** 10.3390/medicina57060627

**Published:** 2021-06-16

**Authors:** Christina Frederiksen, Ole André Solbakken, Rasmus Wentzer Licht, Carsten René Jørgensen, Maria Rodrigo-Domingo, Gry Kjaersdam Telléus

**Affiliations:** 1 Psychiatric Clinic North, Brønderslev Psychiatric Hospital, North Denmark Region, 9700 Brønderslev, Denmark; 2 Department of Clinical Medicine, Aalborg University, 9000 Aalborg, Denmark; rasmus.licht@rn.dk; 3 Department of Psychology, University of Oslo, 0317 Oslo, Norway; o.a.solbakken@psykologi.uio.no; 4 Psychiatry—Aalborg University Hospital, 9000 Aalborg, Denmark; mariarodrigo@rn.dk (M.R.-D.); gdkt@rn.dk (G.K.T.); 5 Department of Psychology, Aarhus University, 8000 Aarhus, Denmark; carsten@psy.au.dk; 6 Psychology, Department of Communication and Psychology, Aalborg University, 9000 Aalborg, Denmark

**Keywords:** affect integration, personality disorders, psychopathology, Affect Integration Inventory, emotional dysfunction

## Abstract

*Background and Objectives:* Emotional dysfunction is considered a key component in personality disorders; however, only few studies have examined the relationship between the two. In this study, emotional dysfunction was operationalized through the Affect Integration Inventory, and the aim was to examine the relationships between the level of affect integration and the levels of symptom distress, interpersonal problems, and personality functioning in patients diagnosed with personality disorder according to the Diagnostic and Statistical Manual of Mental Disorders, fifth edition. *Materials and Methods:* Within a hospital-based psychiatric outpatient setting, 87 patients with personality disorder referred for treatment were identified for assessment with the Affect Integration Inventory and other measures (e.g., the Symptom Checklist-90, Revised, the Inventory of Interpersonal Problems 64 circumplex version, and the Severity Indices of Personality Problems). *Results:* The analyses revealed that problems with affect integration were strongly and statistically significantly correlated with high levels of symptom distress, interpersonal problems, and maladaptive personality functioning. Additionally, low scores on the Affect Integration Inventory regarding discrete affects were associated with distinct and differentiated patterns of interpersonal problems. *Conclusion:* Taken together, emotional dysfunction, as measured by the Affect Integration Inventory, appeared to be a central component of the pathological self-organization associated with personality disorder. These findings have several implications for the understanding and psychotherapeutic treatment of personality pathology. Furthermore, they highlight the importance of considering the integration of discrete affects and their specific contributions in the conceptualization and treatment of emotional dysfunction in patients with personality disorders.

## 1. Introduction

As illustrated in the diagnostic conceptualization, emotional dysfunction is considered a central component in personality disorder (PD). In the alternative model of PD (AMPD) presented in section III of the Diagnostic and Statistical Manual of Mental Disorders, fifth edition (DSM-5) [1] and in the upcoming 11th edition of the International Classification of Diseases (ICD-11) [2] evaluations of global severity are emphasized along with ratings of trait facets or trait qualifiers. One essential aspect of personality functioning contributing to severity determination is the manifestation of emotional dysfunction, including the range and appropriateness of emotional experience and expression, the tendency to be emotionally over- or under-reactive and the ability to recognize and acknowledge unwanted emotions [3].

However, emotional dysfunction represents a multifaceted term and has been conceptualized in a variety of ways: alexithymia [4,5], affect integration [6,7], emotion regulation [8], emotional awareness [9], mentalized affectivity [10,11], and emotional intelligence [12].

In the present study, emotional dysfunction was conceptualized and operationalized as affect integration or affect consciousness (AI), which represents a structured approach for understanding the impact of discrete affects on psychological functioning [13]. AI is defined as the capacity for utilizing one’s affect states for adaptive purposes [7,14,15]. AI has been extensively studied and its conceptual relationships with other related constructs have been detailed elsewhere (see e.g., [13,15]). 

In the AI model, different affects are defined as biologically founded responses. However, emotional processes are also subject to learning and socialization, and as a consequence of the individual’s unique developmental history, these processes will organize and become automatized as subjective patterns or scripts for experiencing, comprehending, and expressing one´s affective reactions [15,16,17]. The semistructured Affect Consciousness Interview (ACI) [18] and the self-reported Affect Integration Inventory (AII) [19] have been developed to assess the level of adaptiveness of the individual´s affect organization. Previous research has demonstrated good psychometric properties and clinical usefulness of both the ACI [13,20,21,22,23,24,25,26] and the AII [19,27,28].

### 1.1. The Relationship between Emotional Dysfunction and Severity of Psychopathology in Individuals with Personality Disorders

The relationships between AI and measures of psychopathology have been demonstrated in numerous investigations. Studies based on clinical outpatient samples with high prevalence of personality disorder found that low levels of AI were related to higher levels of psychopathology, interpersonal problems, symptom distress, and overall level of personality problems [6,22]. In a study by Lech et al. [24] based on a mixed sample of nonclinical respondents (*n* = 27) and patients seeking psychiatric treatment for either eating disorder (*n* = 47), relational and social problems (*n* = 13), or stress-related problems (*n* = 8), the results revealed that the clinical groups had significantly lower levels of AI. Furthermore, low levels of AI have been associated with increased symptom distress, interpersonal problems, and more disturbed self-image [24].

However, findings in this area have not been entirely consistent, e.g., in a study with a sample of individuals with PD, Normann-Eide et al. [23] found that low levels of AI measured by the ACI were associated with higher levels of interpersonal problems and lower levels of self-esteem but not with higher levels of symptom distress or the number of fulfilled criteria measured by Structured Clinical Interview for DSM-IV Axis II Personality Disorders (SCID-II) [29]. 

Other studies have addressed the impact of emotional dysfunction by examining the relationship between psychopathology and alexithymia, which is a deficit in the processing of emotional experiences manifested by difficulties in identifying and distinguishing feelings from somatic sensations, difficulties in verbalizing feelings, a restricted fantasy life, and an externally oriented style of thinking [4]. In a sample of 388 patients with different PDs, Nicoló et al. [30] measured levels of alexithymia with the Toronto Alexithymia Scale 20 (TAS-20) [4] and examined the relationships between alexithymia and diagnostic features, symptom distress, and interpersonal difficulties. The results revealed that high levels of alexithymia were related to higher levels of PD traits, especially cluster C traits [1], more severe symptom distress, and interpersonal problems [30]. These findings were also in line with a study by Bach et al. [31], who found that dependent, avoidant, and schizotypal personality dimensions and lack of histrionic dimensions were related to the presence of alexithymia.

Several studies have linked the level of emotional dysfunction in cluster C PDs to higher severity of psychopathology. In regard to other PDs, a meta-analysis by Derks et al. [32] found moderate to strong positive associations between (a lack of) emotional awareness and borderline PD. Furthermore, borderline PD and impairments in interpersonal functioning and impulsivity have repeatedly been linked to mentalizing deficits [33], mindfulness deficits [34], emotional dysregulation [35], and low emotional intelligence [36].

Johansen et al. [26] addressed the relationship between emotional dysfunction (measured by the ACI) and maladaptive personality function as operationalized through the Severity Indices of Personality Problems (SIPP-118) [37]. In a sample of patients diagnosed with either borderline PD or avoidant PD, Johansen et al. [26] found significant relationships between low levels of AI and more severe problems with identity integration and relational disturbance.

In summary, despite some inconsistency, it appears that the level of emotional dysfunction in individuals with PD is linked to the severity of psychopathology. However, the scientific bases of our knowledge of the issue are still rudimentary and in need of further elaboration and clarification.

### 1.2. The Present Study

Considering the increased focus on fundamental dysfunctions in the adaptive management of affects in PDs, the aim of this study was to examine associations between the level of emotional dysfunction as assessed by the AII and the severity of psychopathology in individuals with PDs. Specifically, we address associations between AI and symptom distress, interpersonal problems, and various aspects of personality functioning. To the best of our knowledge, this is the first study to systematically examine these relations in this way in a clinical sample of patients with PD. Based on theoretical assumptions and previous studies (e.g., [19]), we expect to find associations between low levels of AI and more pronounced symptom distress, interpersonal problems, and maladaptive personality functioning. The study will test the following hypotheses:

(1) Problems with AI are associated with symptom distress and overall relational difficulties.
(2) Problems with AI for discrete affects are systematically and predictably associated with specific types of interpersonal problems (see the statistical section for further elaboration of the expected relations on page 5).
(3) Problems with AI are associated with the severity of personality dysfunction.

## 2. Materials and Methods

### 2.1. Participants

The current study is based on data collected at two specialized hospital-based outpatient units treating all types of PDs except for schizotypal PD and antisocial PD. Both hospital units are based in the Psychiatric Health Care Services of the North Denmark Region, and deliver psychotherapy in individual, group, or combined settings on a weekly basis for individuals with PD. Patients who were referred to treatment for PDs at the outpatient units, met the inclusion criteria of a diagnosis of PD according to the DSM-5 [1], were above the age of 18, were literate in Danish, and gave informed written consent to participate, were recruited. Patients with comorbid psychotic disorder and bipolar I disorder were treated elsewhere and thus excluded from the study. So were patients with developmental disorder (e.g., Asperger’s disorder), or a diagnosis of drug or alcohol dependence potentially interfering with the outcome measures.

### 2.2. Procedures 

The diagnostics of the patient were assessed by the semistructured Present State Examination (PSE) [38] and SCID-II [29]. The interviews were conducted by experienced psychiatrists, and psychologists who were trained in the use of the instruments. Final diagnostics were determined according to the DSM-5 [1].

The data on symptom distress, interpersonal problems and personality functioning were based on self-reported measures and collected through an online self-administered survey using the platform SurveyXact (Ramboll, Aarhus, Denmark).

All patients were informed that participation was voluntary, and that nonparticipation would not influence their treatment in any way. Written and oral information about the study was provided before recruitment. The study was conducted in accordance with the Helsinki Declaration and approved by the Danish Data Protection Agency on 1 May 2014 (2019-017816). No approval was needed from the Danish National Committee on Biomedical Research Ethics due to the nature of the study.

### 2.3. Measures

#### 2.3.1. Affect Integration Inventory 

The AII [19] consists of 112 statements about perception of awareness, tolerance, and expressions of nine discrete affects: (1) Interest, (2) Joy, (3) Fear, (4) Anger, (5) Shame, (6) Sadness, (7) Jealousy, (8) Guilt, and (9) Tenderness. Items are phrased so they tap into either the Experience or the Expression aspects of the affects, covering all aspects of the AI concept. Eighty-two items are indicators of the capacity of Experience, while 30 items are indicators of Expression. The items are rated on a 10-point Likert scale ranging from *does n*o*t fit at all* (0) to *fits perfectly* (9). Higher scores correspond to higher levels of AI. The scores for this study were computed at three levels: (1) a mean overall Global AI score, (2) a mean score on the capacity of Experience across affects or a mean score on the capacity of Expression across affects, and (3) mean scores for each of the discrete affects. The psychometrics of the AII have been validated in both clinical and nonclinical samples [19,27,28]. The Cronbach’s alpha values from the present sample have been published elsewhere [27]. These were generally high, ranging from 0.70 (Sadness) to 0.94 (Global AI), with a median of 0.83 [27].

#### 2.3.2. The Symptom Checklist-90, Revised (SCL-90-R) 

The SCL-90-R [39] is a well-established 90-question self-reported scale designed to assess psychopathological symptoms. On a 5-point Likert scale ranging from *not at all* (0) to *very much* (4), the intensity of symptoms during the last seven days is rated. The Global Severity Index (GSI) is calculated as an average across all 90 items and serves as an indicator of the current level of general distress. The Cronbach’s alpha for the GSI was 0.95.

#### 2.3.3. Inventory of Interpersonal Problems 64 Circumplex Version (IIP-64) 

The IIP-64 [40] is applied to assess the level of general and specific interpersonal problems. All items are rated on a 5-point Likert scale ranging from *not at all* (0) to *very much* (4). The IIP-64 yields an overall score and eight octant subscale scores. The latter are organized in a circular order constituting the interpersonal circumplex [40]. While the total score (IIP-Global) serves as an indication of the general level of interpersonal problems, each of the eight octant scores represents specific and systematically interrelated types of interpersonal problems: Domineering, Vindictive, Cold, Socially Inhibited, Nonassertive, Overly Accommodating, Self-sacrificing, or Intrusive. The IIP-Global has previously been linked to symptom severity and negative affectivity [41], and the circumplex structure has revealed good construct validity in terms of fit and patterns of convergent-discriminant associations with external correlates [42]. The Cronbach’s alpha values for the sample were 0.90 for IIP-Global, 0.76 for Domineering, 0.72 for Vindictive, 0.80 for Cold, 0.81 for Socially Inhibited, 0.87 for Nonassertive, 0.73 for Overly Accommodating, 0.73 for Self-sacrificing, and 0.73 for Intrusive.

#### 2.3.4. The Severity Indices of Personality Problems 

The SIPP-118 [37] is a self-reported questionnaire that measures core components of maladaptive personality functioning. Applying a dimensional “self-other” perspective, in the assessment of severity of personality dysfunction the SIPP-118 links to the diagnostic approach presented in the DSM-5 AMPD [43]. The questionnaire consists of 118 items that can be converted into 16 facets and organized into five higher-order domains: (1) The Identity Integration Domain covering the experience of coherence of identity and the experience of oneself as stable, integrated, and purposive; (2) The Relational Functioning Domain covering the capacity to build and maintain genuine caring long-term relationships and to communicate personal experiences and engage with the experiences of others; (3) The Self-control Domain covering the capacity to tolerate, use, and control emotions and impulses; (4) The Social Concordance Domain covering the ability to withhold aggressive impulses and the ability to cooperate with others; and (5) The Responsibility Domain covering the ability to set and achieve realistic goals. Each of the 118 statements is rated on a 4-point Likert scale ranging from *I fully disagree*(1) to *I fully agree* (4). Higher scores equal more adaptive functioning. Although previous studies on the psychometrics of the SIPP-118 have not been completely unambiguous [44], three studies have reported good psychometric properties, including cross-national consistency [45,46,47]. The Cronbach’s alpha values for the sample were 0.88 for the Self-control Domain, 0.84 for the Identity Integration Domain, 0.69 for the Responsibility Domain, 0.79 for the Relational Functioning Domain and 0.84 for the Social Concordance Domain.

### 2.4. Statistical Analyses

The demographic variables were summarized as counts and percentages for categorical variables and means and standard deviations for continuous variables. The associations between the GSI and IIP-Global scores as dependent variables and the Global AI, Experience, and Expression scores as explanatory variables were analyzed using separate simple linear regressions (six in total). Pearson correlation coefficients were computed for each of the AII affects and the IIP-64 octants. The eight correlation coefficients computed for each affect were plotted against the IIP-64 octants, and the corresponding theoretical sinusoidal curve from Solbakken [19] was added to the plot. These theoretical curves are as follows: (1) problems with Tenderness and Guilt have a correlation pattern peaking in the Cold octant with a low point in the Self-sacrificing octant; (2) problems with Anger have a correlation pattern peaking in the Nonassertive octant with a low point in the Dominant octant; (3) problems with Jealousy have a correlation pattern peaking in the Vindictive octant with a low point in the Overly Accommodating octant; and (4) problems with Interest, Joy, Shame, Sadness, and Fear have a correlation pattern peaking in the Socially Avoidant octant with a low point in the Intrusive octant. The associations between the AII and the SIPP-118 domains were studied using Pearson correlation coefficients and are shown as heatmaps. Correlation magnitudes were interpreted according to Cohen´s classifications, i.e., coefficients on the order of 0.10 are small, those of 0.30 are medium, and those of 0.50 are large in terms of the magnitude of effect sizes [48]. Z-tests were conducted to assess differences in correlation magnitudes. Missing data were not imputed. Analyses were performed in Stata 14 (StataCorp, College Station, Texas, USA), and results with *p*-values below 0.05 were considered statistically significant.

## 3. Results

### 3.1. Demographic and Clinical Characteristics of the Sample

The demographic characteristics of the 87 participating patients are shown in Table 1. Most of the participants were female (85%) and living in a cohabitation relationship (58%). One in four reported acts of self-harm within the last three months (26%). A substantial subset had additional diagnoses of mood and anxiety disorders (31% and 24%, respectively). The two most common primary axis II diagnoses were avoidant PD (41%) and borderline PD (34%). Axis I co-morbidity in the sample was similar to other comparable studies, with mood and anxiety disorders being the most prevalent.

### 3.2. Relationships between Affect Integration, Symptom Distress, and Relational Difficulties

**Figure 1:**
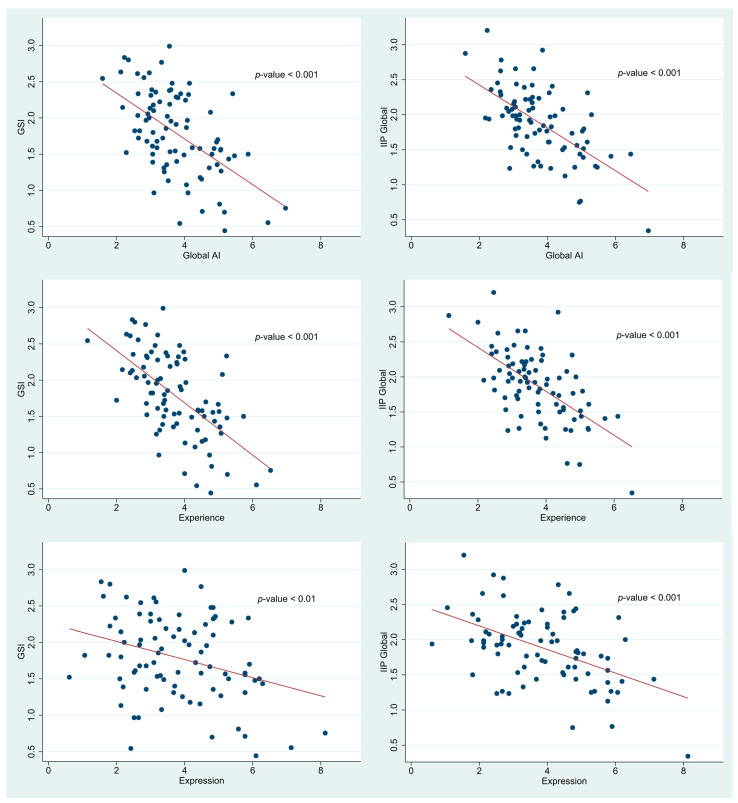
Scatter plots with fitted regression lines (in red) of the associations between the Global Severity Index (GSI) and Affect Integration Inventory (AII) scores (left, *n* = 86) or Inventory of Interpersonal Problems score (IIP-Global) and AII scores (right, *n* = 82). For each pair of variables, the *p*-value from the simple linear regression is shown.

The results revealed that low AI scores were strongly associated with high levels of symptom distress and relational difficulties (see Figure 1). For the GSI, the standardized regression coefficients (correlations) were −0.57 (95% CI [−0.74, −0.38] for Global AI (*r*^2^ = 0.33), −0.61 (95% CI [−0.79, −0.44] for Experience (*r*^2^ = 0.37), and −0.32 (95% CI [−0.52, −0.11] for Expression (*r*^2^ = 0.10). For IIP-Global, the standardized regression coefficients were −0.62 (95% CI [−0.80, −0.44] for Global AI (*r*^2^ = 0.38), −0.61 (95% CI [−0.79, −0.44] for Experience (*r*^2^ = 0.37), and −0.49 (95% CI [−0.68, −0.29] for Expression (*r*^2^ = 0.24).

### 3.3. Patterns of Relationships between AI for Discrete Affects and Specific Types of Interpersonal Problems

**Figure 2:**
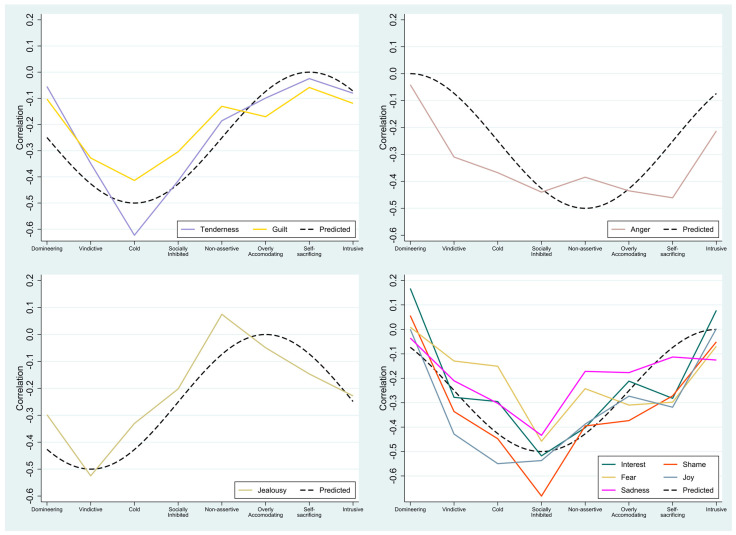
Patterns of relationships between the discrete affects and specific types of interpersonal problems. The predicted patterns are shown as black dashed lines (*n* = 82).

The predicted and observed patterns of associations between AI for discrete affects and specific types of interpersonal problems are presented in Figure 2. The integration of Tenderness and Guilt had patterns of correlations peaking in the Cold octant. For Jealousy, the correlations peaked in the Vindictive octant, whereas for Interest, Shame, Fear, and Sadness, the pattern of correlations peaked in the Socially Inhibited octant. For Joy, a pattern peaking in the Cold octant was obtained. Finally, Anger had a pattern of correlations with its low point in the Domineering octant and its peak in the Self-sacrificing octant.

### 3.4. Relationship between AI and Personality Functioning

**Figure 3:**
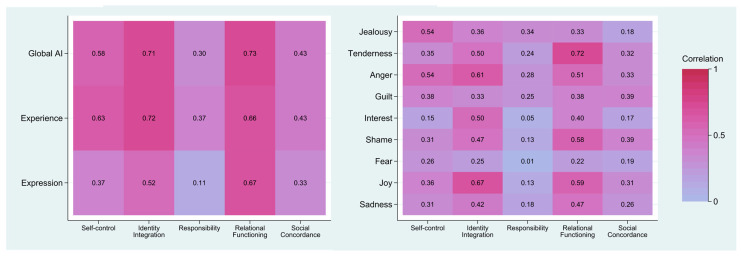
Heatmaps of correlations between SIPP-118 domains and AII scores (left) and discrete affects (right) (*n* = 82).

All correlations between the AII scores (Global AI, Experience, Expression, and discrete affects) and the SIPP-118 domains were positive, ranging from 0.007 (negligible association) to 0.73 (very strong association). As shown in Figure 3 (left), both Identity Integration and Relational Functioning were strongly associated with Global AI, Experience, and Expression. Self-control was strongly associated with Global AI and Experience but moderately associated with Expression. Social Concordance was moderately associated with Global AI, Experience, and Expression. Finally, Responsibility was moderately associated with Global AI and Experience but uncorrelated with Expression.

In the examination of the integration of specific affects, which can be seen in Figure 3 (right), we found that Identity Integration had strong or moderate to strong correlations with Tenderness, Anger, Interest, Shame, Sadness, and Joy, moderate correlations with Jealousy and Guilt, and a weak correlation with Fear. Relational Functioning had strong or moderate to strong correlations with Tenderness, Anger, Shame, Sadness, and Joy, moderate correlations with Jealousy, Guilt, and Interest, and a weak correlation with Fear. Self-control had strong correlations with the integration of Jealousy and Anger, moderate correlations with Tenderness, Guilt, Shame, Joy, and Sadness, and weak correlations with Interest and Fear. Social Concordance had moderate correlations with Tenderness, Anger, Guilt, Shame, and Joy and weak correlations with Jealousy, Interest, Fear, and Sadness. Finally, Responsibility had a moderate correlation with Jealousy, small to moderate correlations with Tenderness, Anger, and Guilt, and small correlations with Shame, Sadness, Joy, and Interest.

## 4. Discussion

The present study examined the relationships between emotional dysfunction and the severity of psychopathology in patients with PDs. Our hypothesis that low levels of AI would be associated with more pronounced symptom distress, more interpersonal difficulties, and higher levels of maladaptive personality traits along with the hypotheses about the relationships between the integration of discrete affects and specific patterns of interpersonal problems were all confirmed. Based on the above results, the Global AI explained 32.5% of the variation in symptoms, 38.4% of the variation in overall interpersonal dysfunction, and between 9.1% (Responsibility) and 53.1% (Relational Functioning) of the variation in personality dysfunction domains. In addition, the integration of discrete affects was associated with distinct patterns of interpersonal problems peaking in separate and expected octants of the interpersonal circumplex.

### 4.1. AI and Symptom Distress

As expected, low levels of AI were strongly correlated with symptom distress (measured by the SCL-90-R). Approximately one-third of the variation in symptoms was explained by the level of AI, thus confirming our first hypothesis. Similar to previous studies [6,22], the results demonstrated a stronger association (Z = 3.76, *p*-value < 0.001) between the Experience aspect of the AI and GSI (−0.61 95% CI [−0.79, −0.44]) than between the Expression aspect and the GSI (−0.32 95% CI [−0.52, −0.11]). This bolsters the notion that dysfunction in the capacity to openly perceive, tolerate, and understand affective experiences is of greater importance to symptom formation than the capacity to directly and clearly express one´s affective states.

Interestingly, the results contrast with previous findings in patients with PD by Normann-Eide et al. [23], who, in a comparable sample of patients with either borderline PD or avoidant PD, did not detect a statistically significant relationship between levels of AI and symptom distress. In particular, two factors might explain this difference in outcome. First, when assessing AI, Normann-Eide et al. [23] applied the ACI. As noted, it has been demonstrated how the ACI and the AII tap into somewhat different dimensions of the AI construct [27]. This leaves the possibility that in a sample consisting of patients suffering from PDs, the association between AI and symptom distress is more easily detectable when assessed with the AII. Second, we note that the sample in the study by Normann-Eide et al. [23] appears to be more homogeneous than ours. It included patients suffering from a primary diagnosis of either borderline or avoidant PD only, whereas our study sample consisted of a wider range of PDs. Restricting the range of the sample will reduce the examined variability in personality functioning, thus increasing the risk of missing substantive associations present in the real world.

### 4.2. AI and General Interpersonal Problems

As hypothesized, low levels of AI were strongly related to more pronounced relational difficulties. Global AI had a correlation of −0.62 with overall interpersonal problems and thus explained close to 40% of its variation, indicating that AI is an essential and highly central feature of interpersonal functioning. Even though experience (*r* = −0.61 95% CI [−0.79, −0.44]) was numerically somewhat more strongly correlated with interpersonal problems than expression (*r* = −0.49 95% CI [−0.68, −0.29]), the correlations were not statistically significantly different (Z = 1.78, *p*-value = 0.07). This result, nevertheless, indicates that the capacity for perceiving, accepting, and reflecting upon affective states is paramount in navigating relationships and even more important to relational health than the capacity to express oneself. Additionally, these results fit well with the considerations of Bateman, Fonagy and Luyten [49], who stressed the interpersonal implications of a deficit in mentalizing capacities.

### 4.3. AI and Specific Types of Interpersonal Problems

The obtained patterns of associations between AI for discrete affects and specific types of interpersonal problems were in overall agreement with our hypotheses. As postulated, four distinct patterns were identified with peaks in separate octants of the interpersonal circumplex. Difficulties with Jealousy corresponded to a relationship profile characterized by interpersonal Vindictiveness. Difficulties with Tenderness, Joy, and Guilt corresponded to a profile characterized by Coldness. Difficulties with Interest, Shame, Fear, and Sadness corresponded to a profile characterized by Social Inhibition. Finally, difficulties with Anger corresponded to a relationship profile characterized by Self-sacrifice and Over-accommodation.

In summary, seven of the nine affects had correlation patterns closely aligned with our expectations. The remaining two affects deviated somewhat from our hypotheses. First, Integration of Joy had a pattern of correlations rotated slightly clockwise in the interpersonal space with its peak in the Cold rather than in the expected Socially Inhibited octant. Second, Integration of Anger was more broadly associated with interpersonal problems than hypothesized. It had correlations in the 0.40–0.50 range throughout the Socially Inhibited, Nonassertive, Overly Accommodating, and Self-sacrificing octants with no clear peak in the pattern, and there were sizable correlations with Vindictiveness and Coldness. Interestingly, this indicates that difficulties with Anger have more wide-reaching or general interpersonal implications than what is common for other affects.

### 4.4. AI and Personality Functioning

Positive correlations were expected between AI on all levels and personality functioning across all five domains of the SIPP-118. In accordance with our hypotheses, the results suggested a close link between low levels of AI and more pronounced personality dysfunction. Higher-order AI scores (Global AI, Experience and Expression) were strongly associated with Identity Integration, Relational Functioning, and Self-control and moderately or moderately to strongly associated with Responsibility and Social Concordance.

In line with findings from Johansen et al. [26], who found an association between lower levels of AI and more severe problems within the areas of Identity Integration and Relational Functioning, the strongest correlations in the present study were also found between AI and these two SIPP domains. Strikingly, more than half of the variation in scores on each of these personality functioning domains was explained by the level of AI. This strongly indicates the centrality of affect and affective dysfunction in personality problems as such.

In the DSM-5 AMPD, the generalized severity of PD is considered as the potential most important predictor of both the concurrent and prospective dysfunction in personality psychopathology. Determining severity comprises the evaluation of self (identity, self-direction) and interpersonal (empathy, intimacy) functioning. Interestingly, Bastiaansen et al. [44] found that most of what is included under the self-component of the personality functioning continuum is captured by the Identity Integration domain of the SIPP-118, and the Relational Functioning domain of the SIPP-118 aligned quite well with the interpersonal-component of the personality functioning continuum. Limited by the study design we cannot address the causality of relationships. Yet, it seems reasonable to assume that AI could constitute as an underlying psychological capacity that influences upon the self and interpersonal components of personality functioning and thereby indirectly impacts on the severity of dysfunction.

These results also corroborate the central theoretical conjectures of the AI model. Monsen & Monsen [13] suggested that as a consequence of a deficient capacity to use affects as conveyers of meaning and a source of information about the motives for behavior in oneself and others, low levels of AI would produce fundamental disturbances in the organization of self-experience and self-boundary formation, with reduced capacity to form mutual relationships with others as a result [13]. Indeed, our findings support this theoretically derived notion of AI as essential for the capacity to perceive oneself as an integrated, stable, and purposeful individual (as operationalized through the Identity Integration domain of the SIPP-118) and for the ability to establish and maintain genuine relationships with others (as operationalized through the Relational Functioning domain).

We also examined the correlations between the integration of discrete affects and personality functioning. To our knowledge, this feature/area has never previously been examined in the literature. A pattern of associations was identified, where the five personality domains were differentially related to the level of integration of the various discrete affects. Problems with Identity Integration were broadly and substantially associated (r > 0.30) with difficulties across all affects except Fear, with difficulties in the integration of Joy and Anger as the strongest contributors. Problems with Relational Functioning were also substantially related to all affects except Fear and had difficulties with Tenderness and Joy as the strongest contributors. Problems with Self-control were substantially related to seven of the nine affects assessed, with difficulties with Anger and Jealousy as the strongest contributors. Problems with Social Concordance were substantially related to five of the nine affects, with difficulties with Guilt and Shame as the strongest contributors. Finally, problems with Responsibility were substantially related to one of the affects, i.e., difficulties with Jealousy. Taken together, it appears that difficulties in the integration of separate, discrete affects are specifically indicative of the various core personality problems characteristic of PDs.

Integration of these affects may therefore constitute a central mechanism related to the development of personality problems. Furthermore, it appears plausible that AI in general and the integration of specific affects in particular may serve as viable change mechanisms to be targeted in the treatment of PDs.

### 4.5. AI as a Core Mechanism in the Development of Symptoms, Personality Pathology and Interpersonal Problems

Overall, our findings demonstrated that AI is located centrally at the intersection of psychological symptom formation, maladaptive interpersonal behavior, and the personality functioning dimensions central in character and personality pathology. Levels of AI accounted for substantial though varying amounts of variation in all these domains, and we may speculate that AI constitutes a core mechanism binding those domains together in a meaningful whole. Our results are thus consistent with the conjecture that failures in the integration of ongoing affective activation (a) contribute to the development of psychological symptoms, (b) give rise to maladaptive interpersonal strategies and behaviors, and (c) become structuralized as character-based, dysfunctional ways of perceiving, interpreting, and reacting to events and people in the world.

This interpretation fits well with the theoretical propositions by Izard [50], Krystal [51], Monsen & Monsen [13], Solbakken, Hansen, & Monsen [15], Stolorow, Atwood, & Brandchaft [7], and Tomkins [16,17] and empirical findings by, e.g., Monsen et al. [6], Solbakken, Hansen, Havik, & Monsen [22], Solbakken, Hansen, Havik, & Monsen [21], Solbakken et al. [19], and Taarvig et al. [20]. We believe that support for this conjecture has not previously been so clearly demonstrated in the specific context of PD pathology as in the present study.

### 4.6. AI as a Mechanism of Change in Psychotherapy

Our findings similarly point to AI as a transtheoretically relevant mechanism of change in psychotherapy for PD and beyond. Its position as substantially and similarly predictive of functioning in most major areas of relevance to the treatment of PDs makes it promising as a focal target of psychological interventions. Previous studies and the current study have demonstrated its consistent associations with symptomatology, interpersonal relatedness, and personality functioning across diverse populations, both clinical and nonclinical, and its potency as a predictor and mechanism of change in psychotherapy [21,26,30,52]. Similarly, treatment directed specifically at improving AI has been shown to reduce symptomatic, relational, and characterological problems in mixed clinical populations, patients with PD, and patients with somatoform disorders [52,53,54,55]. In line with Doss [56], AI may prove to be particularly fruitful when examined as a change mechanism in psychotherapy.

### 4.7. Implications for the Field of Knowledge—Generalizing between AI and Related Constructs of Emotional Dysfunction

In this study, emotional dysfunction was operationalized through the AI construct. It is, however, important to note that the AI model shares substantial conceptual overlap with other theoretical constructs dealing with the processes of emotional functioning, e.g., emotion regulation/difficulties in emotion regulation, alexithymia, and mentalized affectivity [15,57]. Even though there are differences in operationalization and definition across these concepts, they broadly correspond in terms of their overarching construct domains. Thus, we argue that knowledge claims made in the present study can be generalized to other conceptualizations to a substantial degree (at least for the higher-order AI scores).

One related concept that has received substantial attention in the recent PD literature is mentalized affectivity. Mentalized affectivity is defined by a mature capacity for affect regulation, including reevaluation of affects, through understanding the complex representative relationship between past experiences and present perception. Hence, while remaining within the affective state, affect regulation is transformed through reflection [10,11]. Mentalized affectivity has recently been operationalized through the Mentalized Affectivity Scale [58]. It would appear that mentalized affectivity in this operationalization shares considerable conceptual overlap with the AI construct. Particularly, components of Processing and Expressing emotions, which also hold the highest predictive value in differentiating among clinical and nonclinical samples [58], are highly similar to the capacities for Experience and Expression in AI. Taking these conceptual overlaps into consideration, studies on AI seem especially well suited for generalizing to the mentalized affectivity concept and vice versa [11,15,57,58], and the combination and integration of findings in the literature on AI and mentalized affectivity should be particularly fruitful.

## 5. Strengths and Limitations

The present study was based on a well-characterized sample of patients with PDs, including a variety of PD types, thus increasing the generalizability of our results. Additionally, the study and its hypotheses were grounded in a detailed and coherent theoretical model that operationalizes emotional dysfunction, making it possible to test specific hypotheses with a robust conceptual basis. The study conjointly addressed major areas of psychological dysfunction in PDs and allowed us to systematically examine the contribution of AI to all these areas. By applying the recently developed AII, the study contributes a novel method of assessing the relationship between emotional dysfunction and psychopathology. Finally, as the AII yields systematic assessment on the level of discrete affects, the study provides insights into the unique contribution of the integration of these affects, improving our understanding of PD pathology.

Regarding the limitations, all instruments were self-rated, which may inflate associations. Second, the sample size was relatively small. Third, the cross-sectional nature of the study precludes us from empirically addressing any potential causal relationships between the tested variables. Fourth, the diagnostic assessments of the patients were performed by different clinicians, although all were experienced, and the diagnosis of each patient was evaluated at a multidisciplinary diagnostic conference at specialized PD units. Fifth, this study was conducted with a mixed sample of patients suffering from different PDs, so the findings might not apply to specific PD subtypes. Relatedly, one should be somewhat cautious in generalizing to PDs in general since the majority of the sample had either borderline PD or avoidant PD.

## 6. Conclusions

In support of our hypotheses, the results provide evidence on the centrality of affective dysfunctions in PD. Low levels of AI were strongly associated with more pronounced symptom distress, interpersonal difficulties, and maladaptive personality traits. The results also supported the hypothesis that dysfunction in the management of discrete affects is related to specific patterns of interpersonal and personality problems, indicating the importance of considering the impact of discrete affects when treating PDs and associated pathology. In summary, even though no causality can be inferred from our findings, AI appears to be centrally located at the intersection of symptomatic, relational, and characterological dysfunctions common in PDs and may be both a core factor in the development of these problems and a potential target in treatments directed at their alleviation.

Future studies should address the relationship between specific aspects of AI in specific PDs and the severity of psychopathology. We have focused on monitoring the relationship between overall levels of AI and psychopathology. Whether personality dysfunction may be related to particular modes of AI (e.g., over-regulation/under-regulation, acting out/acting in, being driven by/lacking access to the various affects examined) was beyond the scope of this study. Hopefully, future studies will examine these characteristics so that tailored therapeutic interventions targeting emotional dysfunctions can be developed and implemented accordingly.

## Figures and Tables

**Table 1:** Demographic and clinical characteristics of the patient group (*n* = 87).

Age *^,a^	31.7 (9.5)
Sex, female ^+^	74 (85.1%)
Married/cohabiting ^+,b^	49 (57.6%)
Completed high school ^+,b^	31 (36.5%)
Self-harm within the last three months ^+,b^	22 (25.9%)
Suicide attempt within the last three months ^+,c^	<4%
Mood disorder ^+,b^	27 (31.0%)
Anxiety disorder ^+,b^	21 (24.1%)
Substance abuse ^+,b^	4 (4.6%)
Eating disorder ^+,b^	3 (3.4%)
Behavioural disorder ^+,b^	4 (4.6%)
Primary PD diagnosis ^+^	
Borderline	30 (34.5%)
Avoidant	36 (41.4%)
Mixed	15 (17.2%)
Other ^e^	6 (6.9%)
No. of PD-diagnoses *	1.4 (0.6)
GSI *^,a^	1.8 (0.6)
IIP-Global *^,d^	1.9 (0.5)

*: mean(sd); ^+^: count (%). ^a^: information available for 86 patients; ^b^: information available for 85 patients; ^c^: actual count not reported; ^d^: information available for 82 patients. ^e^: the PD-diagnosis group “other” consist of obsessive-compulsive PD, narcissistic PD and paranoid PD.

## Data Availability

The data that support the findings of this study are available from the corresponding author upon reasonable request.
